# Supplementary material for: Development of chemokine network inhibitors using combinatorial saturation mutagenesis
Source: Commun Biol. 2025 Apr 3;8:549. doi: 10.1038/s42003-025-07778-6 (PMC11969024; doi:10.1038/s42003-025-07778-6)
Supplement: Supplementary file 4 — Supplementary Data 2 [file 42003_2025_7778_MOESM4_ESM.zip › SupplementaryData3/scripts/xNNKi.tar!/./script/analysis/JK/heatmaps/HD2Xallheatmap.pdf]

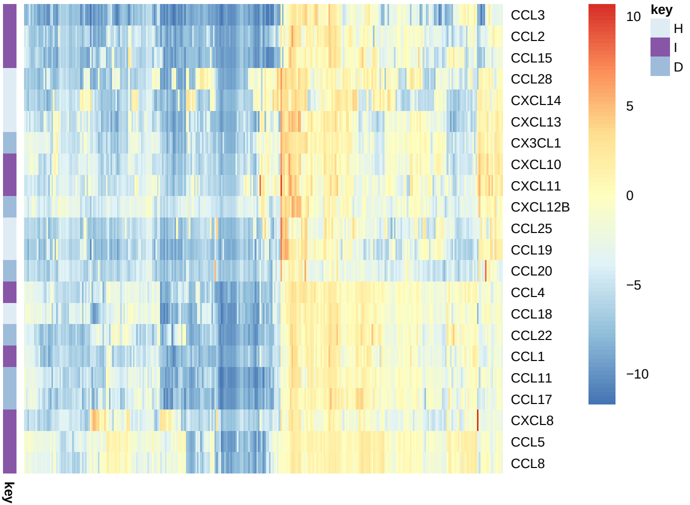
CCL3
CCL2
CCL15
CCL28
CXCL14
CXCL13
CX3CL1
CXCL10
CXCL11
CXCL12B
CCL25
CCL19
CCL20
CCL4
CCL18
CCL22
CCL1
CCL11
CCL17
CXCL8
CCL5
CCL8
10
5
0
−5
−10
key
H
I
D
key
